# Multi-Objective Genetic Algorithm for Cluster Analysis of Single-Cell Transcriptomes

**DOI:** 10.3390/jpm13020183

**Published:** 2023-01-20

**Authors:** Konghao Zhao, Jason M. Grayson, Natalia Khuri

**Affiliations:** 1 Department of Computer Science, Wake Forest University, 1834 Wake Forest Road, Winston-Salem, NC 27109, USA; 2 Department of Microbiology and Immunology, Wake Forest School of Medicine, Medical Center Boulevard, Winston-Salem, NC 27157, USA

**Keywords:** cluster analysis, genetic algorithms, multi-objective optimization, single-cell RNA-sequencing, transcriptomics

## Abstract

Cells are the basic building blocks of human organisms, and the identification of their types and states in transcriptomic data is an important and challenging task. Many of the existing approaches to cell-type prediction are based on clustering methods that optimize only one criterion. In this paper, a multi-objective Genetic Algorithm for cluster analysis is proposed, implemented, and systematically validated on 48 experimental and 60 synthetic datasets. The results demonstrate that the performance and the accuracy of the proposed algorithm are reproducible, stable, and better than those of single-objective clustering methods. Computational run times of multi-objective clustering of large datasets were studied and used in supervised machine learning to accurately predict the execution times of clustering of new single-cell transcriptomes.

## 1. Introduction

Due to technological advances of single-cell RNA sequencing (scRNA-seq) and spatial imaging, it is now possible to measure the gene expression (transcriptome) of individual cells and catalog their types and states. These catalogs can be used to discover biomarkers to diagnose, treat, and monitor human diseases and disorders [1]. The identification of cell types is based on the prior knowledge about marker genes, which are preferentially expressed in specific cells, tissues, or diseases. However, not all type-specific markers are known, and cell-types must be derived directly from the distributions of gene expression in the sequenced cells. The main idea is to cluster the transcriptomes of individual cells into groups, such that the gene expressions of cells within each group are similar to each other, and dissimilar to the gene expressions of cells in other groups. Cluster analysis has become an important tool for the discovery of common and rare cell types, and for the study of single-cell transcriptomes. Many clustering algorithms have been developed for scRNA-seq data. They can be divided into partitional [2,3,4,5,6,7], hierarchical [8,9,10,11,12,13], graph-based [14,15,16,17,18], and density-based [19,20].

Graph-based approaches use community-detection algorithms, which were originally developed for the analysis of graphs and social networks. Community-detection algorithms focus on the structure of the network as a function of connectivity between cells. These algorithms optimize modularity criterion, which measures the strength of the network communities. Community-detection algorithms have been widely used in scRNA-seq data analysis and they have been incorporated into popular software packages, such as PhenoGraph [21], Seurat [18] and Scanpy [22]. PhenoGraph and Seurat use the Louvain method of community-detection [23], in which they first focus on finding small communities of cells by optimizing local modularity. Next, a new network is formed using the small communities as nodes, and the second round of community detection is performed. Scanpy is based on the Leiden algorithm [17], which addresses one of the limitations of the Louvain method that leaves unconnected communities, by randomly breaking large communities into smaller ones during the optimization. Although popular, community-detection methods tend to overestimate the number of clusters and miss small communities [24].

**Figure 1:**
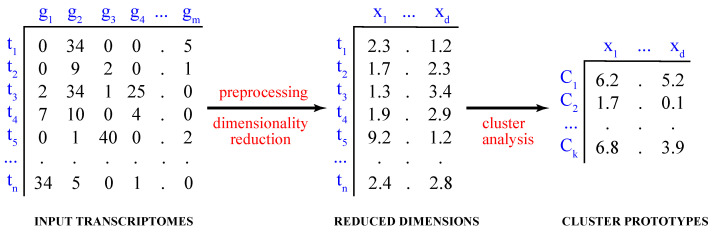
**Cluster analysis of single-cell transcriptomes**. Shown are the steps of cluster analysis of high-dimensional single-cell transcriptomes, including data preprocessing and dimensionality reduction.

Computationally, the process of partitioning the data into distinct groups is known as the partitional cluster analysis. Mathematically, given a dataset of single-cell transcriptomes $T=\{\vec{t_{1}},\vec{t_{2}},\vec{t_{3}},...,\vec{t_{n}}\}$, where $t\in R^{n}$, the objective is to find a set of *k* clusters, $C=\{c_{1},c_{2},c_{3},...,c_{k}\}$, such that $c_{i}\cap c_{j}=\emptyset$ and ${⋃}_{i=1}^{k}c_{i}=T$ (Figure 1).

In cluster analysis, ground truths are not known, and therefore, the choice of the clustering objectives is somewhat arbitrary. More often than not, there exist conflicting objectives, and a unique clustering solution cannot be found, such that it satisfies different objectives. For example, clustering solutions obtained by minimizing the intra-cluster distances between cells, aim to find compact clusters. However, solutions with highly compact clusters may not separate the clusters very well. By optimizing a different objective criterion, such as the inter-cluster distances between cells, well-separated clusters may be found, albeit with lesser cohesiveness.

**Figure 2:**
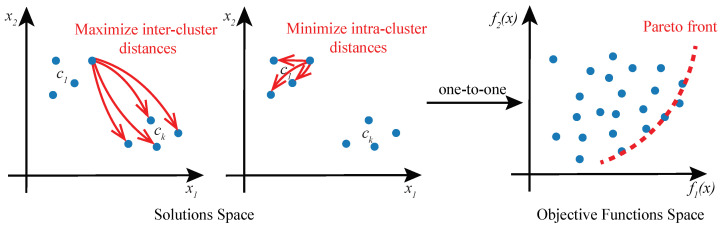
**Cluster analysis as a multi-objective optimization problem.** A solution to a cluster analysis problem is a set of *k* cluster prototypes $c_{1},\ldots ,c_{k}$. Solution space of two objective functions and a corresponding objective function space are shown. Objective function $f_{1}\left(x\right)$ maximizes inter-cluster distances, and $f_{2}\left(x\right)$ minimizes intra-cluster distances. Pareto front encompasses optimal solutions that are not dominated by any other feasible solutions.

Given the existence of multiple objectives in cluster analysis, we propose to approach the problem as a multi-objective optimization (MOO) task and solve it using Genetic Algorithms (GAs). In this formulation of cluster analysis, no single solution exists that can simultaneously optimize all of the multiple objective functions. Rather, the goal of MOO is to find a diverse set of clustering solutions called Pareto-optimal solutions or Pareto front (Figure 2), comprising the best trade-offs between multiple objectives. A solution is said to be Pareto-optimal if no other solution in the solution space can further improve an objective in the objective functions space without degrading other objectives [25]. MOO solution space may be multi-dimensional and it is mapped to the objective function space in a one-to-one relationship.

GAs are well-known heuristics that use the concept of the survival of the fittest, inspired by biological evolution, to search for the best solution to a problem [26,27]. They have been successfully applied in many problem domains to find accurate approximations of solutions of single-objective optimization problems [28,29,30,31,32,33,34]. In GAs, a solution to an optimization problem is encoded in a chromosome, and its fitness value is optimized by the algorithm. GAs iteratively improve a population of chromosomes by applying genetic operators, such as the selection, crossover and mutation. Because GAs work by evolving a population of chromosomes, Pareto-optimal solutions are found via non-dominated solution sorting (NDSS) of chromosomes [35,36]. The final clustering solution is selected from the Pareto front using a user-defined criterion that measures a goodness-of-clustering, for example.

In this work, we design, implement, and validate a multi-objective GA (MOGA) for cluster analysis of single-cell transcriptomes. We assess its performance using 48 experimental and 60 synthetic datasets, ranging from 59 to 10,000 cells, and 2 to 64 clusters. We find that MOGA outperforms alternative clustering algorithms, including community-detection methods, such as Louvain-based PhenoGraph [21] and Seurat [18], and Leiden-based Scanpy [17], in internal and external validation. Moreover, using an innovative metamorphic evaluation of algorithmic stability, we confirm that MOGA produces stable and consistent clustering results. Finally, we train a machine-learning predictor to estimate MOGA’s execution time given the system’s resources and datasets’ sizes.

## 2. Materials and Methods

**Multi-objective clustering:** Given d-dimensional transcriptomes, we aim to find *k* cluster prototypes (cluster centers) by optimizing two objective functions (Equation (1)) as follows.

(1) $$\begin{array}{c} max/min\left\{f_{1}\left(x\right),f_{2}\left(x\right)\right\},\;x\in X,\;X\subseteq R^{d}\\ f_{1}\left(x\right)=\sum_{i=1}^{k}n_{i}\;d\left(z_{i},\bar{z}\right)\\ f_{2}\left(x\right)=\sum_{i=1}^{k}\sum_{x\in c_{i}}d\left(x_{i},z_{i}\right) \end{array}$$

Above, $n_{i}$ is the total number of cells in cluster *i*, *k* refers to the number of clusters, $x_{i}$ denotes a generic cell, $c_{i}$ is the *i*th cluster, $z_{i}$ refers to cluster prototype, $\bar{z}$ is the mean vector of cluster prototypes, and d(x,y) is the Euclidean distance.

The first objective in this formulation is to find well-separated prototypes by maximizing the weighted sum of distances between a cluster prototype and the mean vector of all prototypes. The second objective is to find compact clusters, by minimizing the total sum of distances between cells belonging to the same cluster and the corresponding cluster’s prototype. By optimizing for these two objectives, Pareto-optimal clustering prototypes are found using MOGA.

**Chromosome encoding:** In MOGA, clustering solutions are encoded by the real-valued chromosomes of size l=k×d, where *k* is the number of clusters and *d* is the number of the dimensions of the dataset. Here, *d* denotes the number of Uniform Manifold Approximation and Projection for Dimension Reduction (UMAP) components, derived during preprocessing of the datasets, and it is set to 2. Thus, the first *d* values of a chromosome represent the prototype of the first cluster and the next *d* values represent the prototype of the second cluster, and so on. At the beginning of the evolution, a population of chromosomes is created. Each chromosome is initialized with random values, bounded by the range of values of each dimension of the dataset.

**Evolution:** After the initialization of the population, fitness values of the individual chromosomes are computed, namely $f_{1}\left(x\right)$ and $f_{2}\left(x\right)$, and evolution begins. Parent chromosomes are selected using a tournament selection [37], with the tournament size equal to 20% of the population. Specifically, 20% of chromosomes are randomly chosen from the population pool, and the ones with the best fitness values are selected for breeding. Chromosomes with better fitness values have a greater probability of being selected in the tournament. The tournament selection is repeated to create a population of size *P*, the same size as the initial population.

Next, a one-point crossover is applied with a fixed crossover probability. Its purpose is to exchange the information encoded in the two parent chromosomes and generate two offspring chromosomes. Specifically, for two chromosomes of size *l*, a random integer *i* is generated in the range of [0,l), and the chromosomal parts after *i* are exchanged between the two parent chromosomes. Next, a polynomial mutation with a fixed individual mutation rate is used to select an offspring for mutation. Once an offspring is selected, mutation is performed, with a fixed mutation probability and a crowding value, on every single value of its chromosome. Specifically, the mutation operator generates new floating point numbers within the range of values of each dimension.

**Non-dominance solution sorting:** After the genetic operations of crossover and mutation, the parent population is merged with the offspring population, and NDSS is performed to select chromosomes for the next iteration of the algorithm. NDSS is based on the notion of dominance between solutions. Specifically, one solution dominates another if and only if all objective values of that solution are no worse than the objective values of another solution, and at least one objective value of the solution is better than the other one [38,39]. NDSS may select solutions that are very close to each other. To diversify the population, crowding thresholds are applied to select solutions that are evenly distributed across the entire Pareto front [38,39].

**Selection of the final solution:** In the last step of MOGA, the final clustering solution is selected from the Pareto front. For each Pareto-optimal solution, Davies Bouldin Index (DBI) is computed [40]. DBI measures clustering quality by computing the mean ratio between the intra-cluster and inter-cluster distances over all of the clusters (Equation (2)). DBI values range from 0 to 1, where lower values imply a better clustering quality.

(2) $$\begin{array}{c} DBI=\frac{1}{k}\sum_{i=1}^{k}\max_{j\neq i}\left\{\frac{S_{i}+S_{j}}{d(z_{i},z_{j})}\right\},\;where\;S_{i}=\frac{1}{n_{i}}\sum_{x_{i}\in c_{i}}d\left(z_{i},x_{i}\right) \end{array}$$

Above, *k* is the number of clusters, $n_{i}$ is the total number of cells in cluster *i*, $x_{i}$ denotes a generic cell, $z_{i}$ refers to $i^{th}$ cluster prototype, and d(x,y) is the Euclidean distance between *x* and *y*.

**Hyperparameter tuning:** Population size, number of generations, crowding values, individual mutation rate, mutation probability, and crossover probability are determined by a grid search. Notably, to ensure a fair comparison with baseline methods, including a single-objective GA (SOGA), we tune the parameters for the second objective only, and do not tune the parameters for the first objective (Equation (1)).

Population size and the number of iterations are tuned using the largest experimental dataset with over 6000 single-cell transcriptomes (10X_NCI_A_cellranger3.1). Population size is varied from 100 to 800, and the number of iterations from 1 to 400. The crossover probability, mutation probability, and individual mutation rate are found using a published grid-search protocol [41]. Specifically, the individual mutation rate is varied from 0.3 to 1.0, with a step size of 0.1, mutation probability from 0.0 to 0.1, with a step size of 0.02 and from 0.1 to 0.5, with a step size of 0.1, and crossover probability from 0.5 to 1.0, with a step size of 0.1. The mutation’s crowding values are searched from 0.1 to 1, with a step size of 0.1.

**Evaluation of cluster validity:** Two validations of clustering solutions are performed, namely internal and external validation. In internal validation, ground truths are not known, and the quality of clustering solutions is measured using a widely-accepted internal validity metric, the Silhouette Coefficient (Sil) [42]. Sil is the mean silhouette width of all cells (Equation (3)), where $a(x_{i})$ refers to the mean distance between cell $x_{i}$ from the other cells in the same cluster, and $b(x_{i})$ refers to the minimum of the mean distances of $x_{i}$ from all cells in any other cluster. Sil ranges from −1 to 1, and a higher Sil implies better clustering, with a clear separation and good cohesiveness of clusters. Notably, singletons could exist in clustering solutions, where a single cluster only contains one data instance. Sil handles singletons by setting $S_{i}$ equal to 0, where $b\left(x_{i}\right)=a\left(x_{i}\right)$.

(3) $$\begin{array}{c} Sil=\frac{1}{n}\sum_{i=1}^{n}S_{i},\;where\;S_{i}=\frac{b\left(x_{i}\right)-a\left(x_{i}\right)}{\max\{a\left(x_{i}\right),b\left(x_{i}\right)\}} \end{array}$$

In external validation, cluster prototypes are known, and the validation measures the accuracy of assigned cluster memberships. Two external validity metrics are used, namely the Normalized Mutual Information (NMI) and Adjusted Rand Index (ARI). Similarly to Sil, they are chosen because of their wide adoption by the single-cell transcriptomics’ community. The two metrics are based on different approaches for the evaluation of external cluster validity. ARI uses a pair-counting approach [43,44], by counting pairs of cells that were placed in identical and different clusters. NMI measures the difference in information shared between two clusters and it can be used to compare results with a different number of clusters. Additionally, NMI has been shown to find nonlinear similarities between individual data points [45]. Both NMI and ARI measure the agreement between the predicted and known cluster memberships [46]. The values of both metrics range from 0 to 1, with higher values implying a better agreement between the predicted and known cluster memberships.

NMI is calculated as a ratio of two times the mutual information of two membership lists and the sum of their entropies (Equation (4)), where *I* is the mutual information, *H* is the entropy, and *P* is the probability of a membership label in a list of labels.

(4) $$\begin{array}{c} NMI\left(L,C\right)=\frac{2\times I(L;C)}{H(L)+H(C)},\;where\\ I\left(L;C\right)=H\left(L\right)-H\left(L|C\right),\;H\left(x\right)=-\sum_{i\in x}P_{i}\times log_{2}P_{i} \end{array}$$

To compute ARI, a contingency table of two lists of membership labels is created, tabulating the frequency distribution of the clusters (Equation (5)).

(5) ARI=∑ijnij2−[∑ini2∑jnj2]/n212[∑ini2∑jnj2]−[∑ini2∑jnj2]/n2,

where $n_{ij}$ is the number of times a cell occurs in cluster *i* of labels and cluster *j* of predicted labels at the same index.

**Metamorphic evaluation of clustering stability:** The stability of clustering results is validated using metamorphic perturbations of transcriptomes. The purpose of this validation is to assure that small perturbations of the input data do not change cluster memberships when the perturbed transcriptomes are reclustered [47,48]. Using the scrnabench package (version 1.0), six metamorphic perturbations of the experimental transcriptomes are generated. They include permutation of the order of cells (MR1), modification of counts of a single gene (MR2), duplication of a transcriptome of a single cell (MR3), permutation of the order of genes (MR4), addition of a pseudo-gene with zero-variance expression counts (MR5), and negation of gene counts (MR6). Metamorphic datasets are reclustered and cluster validity metrics are computed and compared with the metrics of the original clusterings.

**State-of-the-art and baseline methods:** MOGA is compared with three state-of-the-art and two baseline methods. To demonstrate the value of multi-objective formulation, MOGA is compared with SOGA, which optimizes only the second objective function (Equation (1)). Therefore, NDSS and DBI-based selection are not performed, and the evolution of SOGA consists of the initialization, fitness evaluation, tournament selection, crossover, and mutation. The final solution of SOGA is encoded by a chromosome with the best fitness value in the last iteration of the algorithm. Notably, all hyperparameters of MOGA are set to the same values as the hyperparameters of the SOGA. Both MOGA and SOGA are implemented using the DEAP package (version 1.3.1) [49].

Additionally, we compare MOGA with another prototype-based clustering algorithm, KMeans, implemented in the scikit-learn package (version 1.1) [50]. All parameters are set to their default values except for the number of iterations, which we set to 350, to match to the number of MOGA and SOGA iterations. PhenoGraph is the state-of-the-art method designed for cluster analysis of single-cell transcriptomes. We use Python’s implementation of the algorithm from the PhenoGraph package (version 1.5.7) [21]. All parameters are set to their default values. Louvain-based Seurat is ran with default parameters using the Seurat package written in R [18], and Leiden-based Scanpy is executed using the Scanpy package written in Python [22].

The number of clusters, *k*, in MOGA, SOGA, and KMeans are set as follows. In external validation, *k* is known and is used as a parameter to the three algorithms. In internal validation, the number of clusters for MOGA, SOGA, and KMeans are set to $0.3\times k_{ph}$, where $k_{ph}$ is the number of clusters that are automatically detected by the PhenoGraph package.

**Datasets:** Two types of single-cell transcriptomic datasets are used, namely experimental and synthetic. These datasets vary in size, sparsity, dimensionality, quality, and the availability of known cluster memberships.

To perform external validation, 60 synthetic datasets are simulated using the Splatter package (version 1.20.0) [51]. Datasets of 10 different sizes are created, ranging from 1000 to 10,000 single-cell transcriptomes, and for each dataset’s size, the number of clusters varies from 2 to 64. Therefore, the true prototypes of these 60 datasets are known.

In internal validation, 48 experimental transcriptomes are used. They comprise transcriptomes of two cell lines, sequenced in 4 centers, using 3 technologies and 2 protocols. The two cell lines are derived from breast cancer (cell line A) and normal B lymphocytes (cell line B). The number of sequenced cells ranges from 59 to 6097, and the number of genes ranges from 16,931 to 32,502, respectively. The average dataset’s sparsity is 78.2% with a maximum of 85.4% and a minimum of 56.2% of zero-valued counts. The raw and preprocessed reference datasets are freely available in the scrnabench package (version 1.0) [52].

**Data preprocessing:** The same standard preprocessing workflow is used to prepare the experimental and synthetic datasets [18]. Specifically, preprocessing comprises five steps: filtering, highly variable gene selection, transformation, scaling, and dimensionality reduction. In filtering, cells with fewer than 200 expressed genes and genes expressed in fewer than three cells are removed. Additionally, cells with mitochondrial content greater than 10% are filtered out. Moreover, outlier cells and genes are removed. The mRNA counts and gene counts are bounded by $10^{mean\left(log_{1}{}_{0}\left(x\right)\right)\pm 2\times std\left(log_{1}{}_{0}\left(x\right)\right)}$ to ensure that each cell has meaningful expression data, where *x* is the total mRNA count or the total gene count per cell.

During the selection of highly variable genes, a mean–variance relationship inherent in single-cell data is modeled, and the top 2000 genes are selected [53]. Next, gene expression counts are transformed using a logarithmic function and scaled such that the mean gene expression across cells is 0, and the variance is 1. The dimensions of the datasets are next reduced to 10 principal components by performing a principal component analysis [54]. These 10 principal components explain more than 98% of variability in all datasets. Finally, principal components are further reduced to two dimensions by the Uniform Manifold Approximation and Projection (UMAP) [55], and cluster analyses are performed using these two-dimensional datasets.

**Estimation of compute time:** Run-time data are collected during the experimentation, including the datasets’ size, number of clusters, combinations of HPC resources, such as the number of CPUs, number of tasks per node, and the number of CPUs per task. These data are used to train a Random forest regressor [56], an ensemble tree-based algorithm for supervised learning. The accuracy of run-time estimates is validated by training a predictor on the simulated datasets and testing it on the reference datasets. Mean absolute error (MSE) between the estimated and actual values is used to evaluate the accuracy of the predictions.

## 3. Results

In this work, we formulate single-cell clustering as a MOO problem, and design and implement a GA to solve it. Specifically, given a dataset of single-cell transcriptomes and the number of clusters, *k*, cluster prototypes are found by maximizing the inter-cluster distances and minimizing the intra-cluster distances.

**Figure 3:**
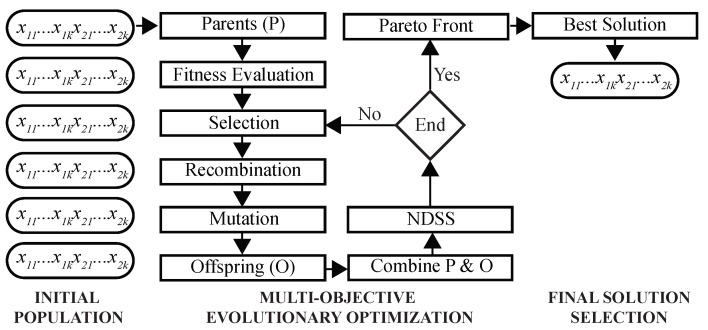
**Architecture of the multi-objective Genetic Algorithm.** Shown are main steps of the proposed GA. Initial population of chromosomes is randomly created and inputted to the GA optimizer. After Pareto-optimal solutions are found, the best solution is selected using a predefined criterion.

This MOO problem is solved using a GA as follows (Figure 3). First, a population of chromosomes is randomly initialized, such that each chromosome is encoded by *k* two-dimensional real-valued prototypes. During the optimization, fitness values of chromosomes are evaluated to select parents for recombination and mutation. Next, NDSS is performed to find clustering solutions on the Pareto front. After the predefined number of iterations is completed, the final solution is selected using DBI as the criterion, and its chromosome is decoded to assign cells to clusters.

**Figure 4:**
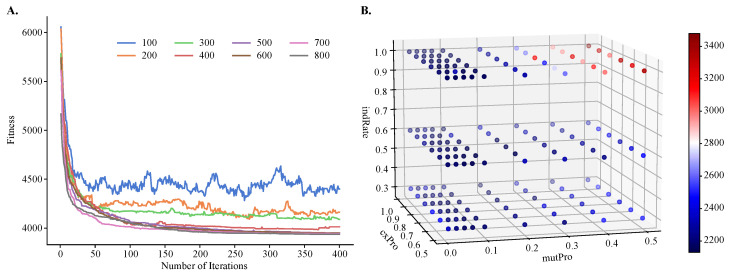
**Hyperparameter tuning of population size, number of iterations, individual mutation rate, mutation probability, and crossover probability for objective $f_{2}\left(x\right)$.** Shown are (**A**) a line plot of fitness values obtained by varying population size and number of iterations and (**B**) a three-dimensional heatmap of fitness values obtained by varying mutation probability, crossover probability, and individual mutation rate. A small fitness value is preferred.

To demonstrate the improvements of MOGA over single-objective clustering methods, in particular over SOGA, we tuned MOGA’s parameters using one objective function only, $f_{2}\left(x\right)$. We found that the algorithm converged after about 50 iterations, regardless of the population size. However, its performance was sensitive to the size of the initial population and the best performance was obtained with larger population sizes (Figure 4A). We also found that GA’s performance depended on the values of the genetic operators, such as the crossover and mutation probabilities and the individual mutation rate. Using a previously published protocol for the grid-search of the parameter space [41], we found that the best performance was achieved with a crossover rate of 0.8, mutation probability of 0.07, and an individual mutation rate of 1.0, respectively. Additionally, the best crowding value for mutation was 0.3, and this parameter controls the degree of similarity between the parents and their offspring.

We first compared MOGA’s performance to the alternative methods on 48 experimental datasets. These recently published single-cell transcriptomic data [57] are publicly available in the scrnabench package [52]. They comprise transcriptomes of two reference cell lines, normal and cancer, which were sequenced by different sequencing centers and technologies and preprocessed using different tools. Therefore, they are suitable to be used as the reference benchmarks for the development of new bioinformatics methods.

**Figure 5:**
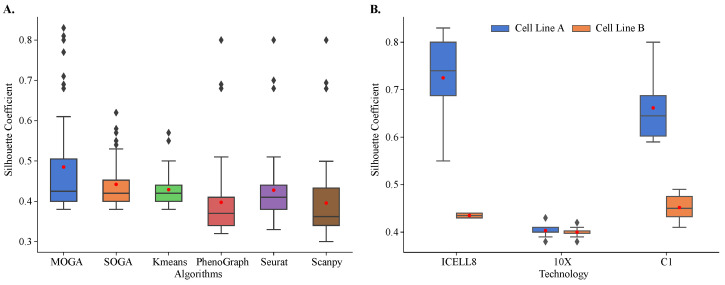
**Internal validation and comparison of MOGA, SOGA, KMeans, PhenoGraph, Seurat, and Scanpy.** Shown are the box plots of (**A**) Sil of 48 scRNA-seq reference datasets with six algorithms and (**B**) Sil scores of MOGA-based clustering by sequencing technology. Experiments are repeated 30 times, and the best Sil values of each dataset are shown. Cell line A: breast cancer; Cell line B: normal B lymphocytes.

Our results showed that MOGA outperformed the state-of-the art and baseline methods in clustering of 48 reference datasets (Figure 5A). These results were reproducible. We repeated cluster analysis 30 times and observed that the results of all algorithms were consistent, and the standard deviations of Sil scores were small. On average, Sil scores were 0.485 ± 0.132 for MOGA and 0.442 ± 0.059 for SOGA. Sil scores of PhenoGraph clustering of these reference datasets were similar to the previously published results. Specifically, Sil scores were below 0.5 for transcriptomes sequenced using 10X and around 0.5 for C1 and ICELL8 datasets [58]. The performance of the Leiden-based community-detection method implemented in Scanpy was on par with PhenoGraph.

Both evolutionary algorithms outperformed PhenoGraph (0.398 ± 0.098) and Scanpy (0.396 ± 0.099), with a statistically significant difference in mean values. On average, SOGA was no worse than KMeans (0.429 ± 0.045) and Seurat (0.428 ± 0.088), while outperforming them in some of the reference datasets, and MOGA significantly outperformed KMeans and Seurat across multiple reference datasets.

When Sil scores of individual reference datasets were considered, striking differences in the performance of different algorithms were observed. The distribution of Sil scores of KMeans was very tight, with most of the scores near the average. In KMeans clustering results, we found two outlier datasets with Sil scores between 0.55 and 0.6. There were more outliers in the SOGA results than in KMeans, and their Sil scores ranged between 0.55 and 0.65. We identified the same three datasets among outliers in PhenoGraph, Seurat, and Scanpy clustering results. Their Sil scores were well above 0.65.

MOGA had the best performance in clustering of the reference datasets. In 12 out of 48 datasets (25%), MOGA had significantly better Sil scores than SOGA, KMeans, PhenoGraph, Seurat, and Scanpy (Table 1). Interestingly, MOGA significantly outperformed other algorithms on transcriptomes that were sequenced using ICELL8 and C1 platforms. Moreover, clustering performance differed for the datasets of the two cell lines. Sil scores of MOGA were significantly higher for the cell line derived from breast cancer (cell line A), which were sequenced using ICELL8 and C1 technologies (Figure 5B). These results were significantly better than those reported previously. The previously reported Sil scores were between 0.5 and 0.6 [57,58], whereas MOGA was able to find better solutions with Sil scores above 0.8 for some of these datasets. On the other hand, Sil scores of clustering of 10X transcriptomes were around 0.4 for both cell-lines, which was observed in previously published results, which used Seurat to cluster cells.

While KMeans, PhenoGraph, Seurat, and Scanpy rely on random initialization and tie-breaking, SOGA and MOGA make significantly more random decisions during the optimization, such as during the crossover and mutation, for example. Therefore, we performed a rigorous metamorphic evaluation of the stability of clustering results [48]. We clustered each of the 48 transcriptomes in their original format and in each of the six metamorphic formats. These metamorphic perturbations included permuting the order of cells (MR1), modifying the counts of a single gene (MR2), duplicating one cell (MR3), permuting the order of genes (MR4), adding a pseudo-gene with zero-variance expression counts (MR5), and negating gene counts (MR6). We computed distributions of Sil scores and used paired T-tests to compare these distributions with the original Sil distributions.

**Figure 6:**
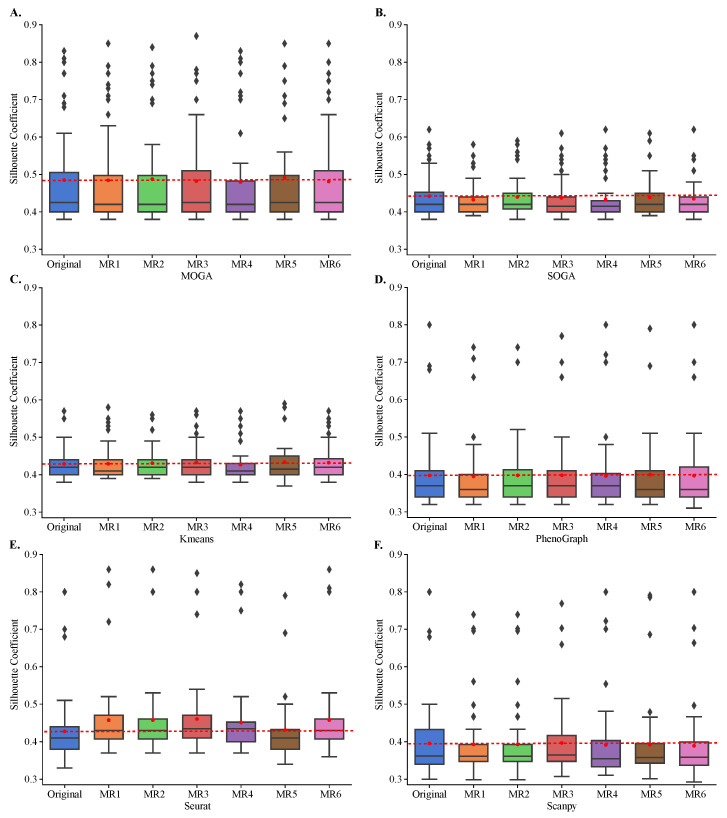
**Cluster stability in metamorphic testing.** Shown are the box plots of the distributions of the Sil of (**A**) MOGA, (**B**) SOGA, (**C**) Kmeans, (**D**) PhenoGraph, (**E**) Seurat, and (**F**) Scanpy in original clustering as well as in metamorphic tests (MR1 to MR6). Experiments are repeated 30 times, and the best Sil of each dataset are shown.

All algorithms, including MOGA, were stable and Sil scores of clustering with metamorphic datasets did not statistically differ from those obtained by clustering of the original datasets (Figure 6). The results were reproducible across 30 repeated experiments of each metamorphic perturbation. Moreover, we confirmed that KMeans tends to perform similarly regardless of the dataset, and achieves Sil scores of around 0.4. We also confirmed that SOGA was able to find better clustering solutions than KMeans, for some of the datasets, with Sil scores reaching 0.6 for a few individual datasets. PhenoGraph, Seurat, Scanpy and MOGA, on the other hand, found better clustering solutions for several datasets, including solutions that had Sil scores near 0.9.

**Figure 7:**
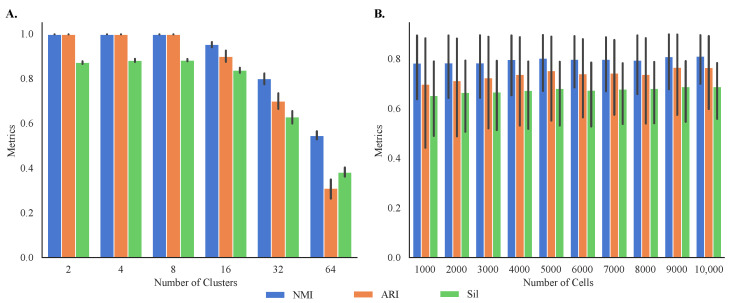
**Internal and external validation of MOGA on synthetic datasets.** Shown are the bar graphs of NMI, ARI, and Sil of (**A**) 60 synthetic datasets with different number of cells and the same number of clusters and (**B**) 60 synthetic datasets with different number of clusters and the same number of cells. Experiments are repeated 30 times, and the best metrics were retained.

Given the differences in performance in clustering of different datasets, we simulated 60 synthetic single-cell transcriptomes with known cluster memberships to better study the sensitivity of MOGA to the characteristics of the datasets, such as their size and the number of clusters. This allowed us to evaluate both internal and external cluster validity. Our results demonstrate that MOGA found accurate cluster prototypes regardless of the datasets’ size (Figure 7A). Specifically, average external validity indices of 60 synthetic datasets, NMI and ARI, were above 0.85 ± 0.163 and 0.77 ± 0.277, respectively. The average external validity index, Sil, was above 0.72 ± 0.205, higher than the average Sil score of experimental datasets.

Our results also indicate that MOGA’s performance was influenced by the number of clusters within the datasets. Specifically, MOGA had an excellent performance when the number of clusters ranged between 2 and 8, and the number of cells ranged between 1000 to 10,000, respectively (Figure 7B). For all dataset sizes, NMI and ARI scores were 1.0 when the numbers of clusters were 2, 4, and 8. When the number of clusters increased to 16, NMI and ARI scores dropped to 0.95 ± 0.02 and 0.90 ± 0.04, respectively. Prediction accuracy for the larger numbers of clusters was lower. For example, NMI values were 0.80 ± 0.04 for 32 clusters and 0.55 ± 0.03 for 64 clusters, respectively. Interestingly, we observed greater disagreements between NMI and ARI scores for 64 clusters. The ARI score for 64 clusters was 0.31 ± 0.07, compared with the NMI score of 0.55 for the same number of clusters. Similar trends were observed for the internal validity metric, Sil. Sil scores were high for the datasets with fewer than 32 clusters (above 0.83 ± 0.02). Average Sil scores for the datasets with 32 and 64 clusters dropped to 0.63 ± 0.05 and 0.38 ± 0.04, respectively.

We point out that both internal and external validity scores of MOGA were on par or better than the scores of alternative methods on the same datasets. For reproducibility, we repeated each experiment 30 times and observed that the standard deviations of all metrics were small, demonstrating good consistency of the results, despite the stochastic nature of MOGA and SOGA.

**Figure 8:**
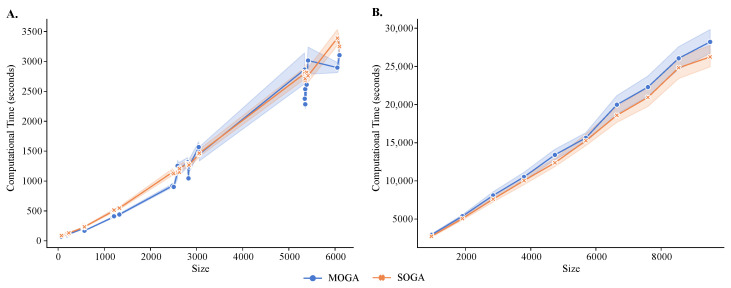
**Running time analysis of MOGA and SOGA.** Shown are time comparisons of MOGA and SOGA (**A**) with 48 scRNA-seq reference datasets and (**B**) 60 synthetic datasets. Experiments are repeated 30 times, and the average computational time is retained.

MOGA had longer execution times than SOGA (Figure 8), and both algorithms had longer execution times than the alternative methods. The average execution time of SOGA was 867.86 seconds (s) for the reference datasets and 5102.99 s for the synthetic datasets, whereas the average time of MOGA clustering was 923.79 s for the reference datasets and 5506.43 s for the synthetic datasets. An exponential relationship existed between the number of instances and the computational time required to cluster the transcriptomes. As the number of instances increased, the number of basic operations in the fitness-value evaluation increased, causing an increase of computational time. These results were relatively stable in 30 repeated experimental runs, although the standard deviations increased as the size of the dataset grew.

While the size of the dataset was directly related to run times, there were other contributing factors, such as the homogeneity of single-cell transcriptomes, for example. For instance, MOGA and SOGA required significantly longer execution to cluster datasets with high heterogeneity, compared with more homogeneous datasets with the same number of cells.

To address the high computational burden of MOGA, we experimented with different combinations of resources on a High Performance Computing (HPC) cluster and parallelized the computation of MOGA objectives using Python’s multiprocessing utilities. The experiments were repeated with six different numbers of CPUs and nodes: 1, 16, 32, 64 (two nodes), and 128 (three nodes). The execution time decreased with the additional CPUs and stopped improving beyond 32 CPUs. In addition, it was clear that with multi-processors, compared with a single processor, the rate of the increase of computational time became smaller. The changes of execution times were negligible when we varied the number of tasks per node and the number of CPUs per task, while keeping the number of CPUs to 32. Therefore, we found the best combination of computational resources for MOGA to be thirty-two CPUs, with four tasks per node and eight CPUs per task.

To put these findings into a practical predictor of run time, we trained a Random forest regressor using 432 execution logs, comprising information about datasets’ sizes, numbers of clusters, and numbers of CPUs and the execution times, which we collected from MOGA experiments on synthetic datasets. We then tested the regression model on the reference datasets, and compared the predicted and the actual execution times. We found that the MSE of predicted run times was about a minute (46.79 s). Thus, the trained ML regressor may be used to estimate computational run times that are needed for clustering of new datasets, given the system’s resources and the dataset’s size.

## 4. Discussion

Single-cell RNA sequencing technologies and bioinformatics tools for their analyses have matured in recent years. Their sensitivity and accuracy hold promise for personalized medicine, as new biomakers of diseases and responses to treatments are being discovered using single-cell transcriptomics [59,60]. Central to these discoveries is the identification of cell types and cell states. Cluster analysis, an unsupervised ML technique, has become a method of choice for cell type identification.

Selection of the most appropriate clustering algorithm for scRNA-seq data analysis remains challenging for the end-users and bioinformaticians. Correctness of the results is a main concern, and their verification is difficult or infeasible because the true cell-type labels are often not known. Additionally, the interpretation of correctness of clustering results depends on an arbitrary choice of the clustering objective, which is used to partition the data. However, cluster analysis is inherently multi-objective because its criteria are ill-defined [61], and the first contribution of this work is the formulation of the prototype-based transcriptome clustering as a MOO problem, solvable using GAs.

Interestingly, applications of MOO in single-cell transcriptomics are limited and mostly used to find the best combination of hyperparameters for deep learning models [62] and to impute missing values [63,64]. For instance, dropouts in single-cell transcriptomics are notoriously difficult to resolve. When unresolved, they propagate noise to downstream analyses and lead to low-quality clustering results. By taking into account topological relationships between cells, the construction of cell–cell affinity matrices can be formulated as a MOO problem [64]. The structure learned from these matrices reduces gene expression noise and improves downstream results. This approach has been recently extended to simultaneously learn three different structures within the raw and noisy data [63]. Specifically, gene–gene similarity matrix, cell–cell similarity, and the low-rank approximation of the data were jointly learned by tuning the parameters of these matrices using GAs. In another application of hyperparameter tuning, evolutionary MOO has been used to simultaneously evolve the hyperparameters and architectures of a deep network of architectures [62].

Most directly related to our work is the use of particle swarm optimization for the scRNA-seq data clustering [65]. Each cell was represented as a particle and raw data were embedded onto multiple subspaces, with each subspace being clustered separately, using a multi-objective particle swarm optimization algorithm. Comparisons with baseline and state-of-the-art methods have been conducted for experimental datasets. The results showed slight improvements over the state-of-the-art method, PhenoGraph. However, only nine datasets were used, and the largest dataset comprised only 5000 cells. In this work, instead of solving a clustering problem by evolving each cell independently, we evolve clustering solutions, and demonstrate an improved performance of our approach using 48 reference datasets and 60 synthetic datasets, ranging between 59 and 10,000 transcriptomes.

Other approaches can be also used to find solutions that balance the trade-offs between multiple objectives. One approach is to optimize one objective function, while constraining the other objective functions [66]. Alternatively, multi-objective optimization can be transformed into a single-objective optimization by aggregating objective functions using a weighted sum. Optimal solutions can be updated via weight changes to find the best weighted sum. However, it has been shown that neither method guarantees that an optimal solution can be found [25], and the two approaches are highly dependent on the chosen constraints and weights.

The main advantages of the MOO formulation of clustering and the proposed MOGA are demonstrated in better performance in the cluster analysis of 48 recently published reference datasets. In 12 out of 48 datasets, MOGA found significantly better results than the original publication [57,58], in particular in datasets of a cancer cell line (cell line A) sequenced using C1 and ICELL8 technologies. Notably, MOGA’s results on 10X dataset are similar to those reported previously and those found by the KMeans, PhenoGraph, Seurat, and Scanpy. These results are interesting, in particular because MOGA outperformed the state-of-the-art PhenoGraph, Seurat, and Scanpy, which were developed specifically for scRNA-seq cluster analysis [17,18,21]. Visual examination of these 12 datasets confirmed the advantage of MOO for scRNA-seq data, whose underlying structure is unknown prior to clustering. These 12 datasets, unlike the other reference datasets, had two well-separated clusters of cells, and these two big clusters were well captured by MOGA. KMeans, SOGA, and community-detection methods, such as PhenoGraph, Seurat, and Scanpy, optimize for cohesiveness or modularity. MOGA, on the other hand, optimizes for both separation and cohesiveness (Equation (1)) and achieves better results on these 12 datasets.

In the second contribution of this study, we confirmed the accuracy, stability, and consistency of MOGA solutions and rigorously compared them to solutions found by KMeans, PhenoGraph, and SOGA. Cluster stability analysis is rarely performed in scRNA-seq studies. Due to the lack of a principled mechanism for the verification of the correctness of the clustering result, two validation techniques are typically used, namely external and internal validation. While these validation methods have convenient metrics for the comparison of new clustering algorithms, end-users may be more interested in clustering results that are meaningful and useful to their specific application domain. End-users may have specific expectations about changes in the clustering results in response to changes in the dataset. For example, a common expectation is that clustering results improve after the removal of noise or after data imputation, or that clustering results should not change if the order of the transcriptomes is permuted. In these scenarios, it is important to assure algorithmic stability of new clustering methods. In this work, we presented and utilized an orthogonal approach to the evaluation of internal and external cluster validity, namely metamorphic evaluation [47,48], and demonstrated that MOGA produced stable and reproducible clustering results.

The design and the implementation of MOGA involved a grid-search for the best hyperparameters, and several decisions had to be made about encoding and genetic operators. In this study, we decided upon the most intuitive design choices, such as real-valued encoding of the chromosomes, tournament and NDSS operations, a one-point crossover, and a polynomial mutation. These choices were most suitable to our application domain; however, many variants of selection, crossover, and mutation exist [67], which can be introduced into future versions of our MOGA. During hyperparameters’ tuning, while we did not observe any dependency between the dataset’s size and performance, we noted that internal and external validity metrics depended on the number of clusters for all algorithms, including MOGA. Specifically, we observed lower ARI, NMI, and Sil scores when the number of clusters changed from 16 to 64. Visual examination of clustering results of these synthetic datasets showed greater overlaps between clusters compared with synthetic datasets with fewer clusters. To handle datasets with overlapping clusters, future extensions of MOGA will involve support for fuzzy clustering [68].

Some limitations of MOGA involve the high computational burden and the propensity of GAs to be trapped at local minima, thus finding optimal solutions for a subset of transcriptomes only. Premature convergence of GAs is a well-known limitation [69,70], which can be addressed in the future versions of MOGA, using adaptive adjustment of the mutation probability, for example. The second limitation of MOGA is its computational speed, which we were able to partly remedy by the parallel evaluation of fitness functions of individual chromosomes. We also timed MOGA’ execution times on an HPC cluster to better understand the dependency between the execution time and system’s resources. In our third contribution, we used these data from the run-time analyses to train a supervised ML estimator of MOGA’s execution times for a given combination of resources and dataset sizes. Our ML-based estimator has a small error of 46.79 s, and can be used to plan future experiments.

In summary, our three contributions demonstrate that the multi-objective cluster analysis of single-cell transcriptomes may be accurately and robustly performed using Genetic Algorithms, and its computational run-times may be predicted based on the system’s resources and datasets’ sizes. This work opens up new directions for the practical applications of multi-objective cluster analysis of single-cell transcriptomes to better guide biomarkers’ discovery.

## Figures and Tables

**Table 1:** **Internal validity of MOGA, SOGA, KMeans, PhenoGraph, Seurat, and Scanpy.** Shown are Silhouette scores of clustering of 12 reference transcriptomes, where MOGA outperformed other methods.

Dataset	MOGA	SOGA	KMeans	PhenoGraph	Seurat	Scanpy
**C1_FDA_HT_A_featureCounts**	0.60	0.55	0.55	0.51	0.51	0.50
**C1_FDA_HT_A_kallisto**	0.59	0.48	0.47	0.43	0.45	0.43
**C1_FDA_HT_A_rsem**	0.61	0.55	0.55	0.47	0.47	0.45
**C1_LLU_A_featureCounts**	0.68	0.54	0.5	0.67	0.68	0.68
**C1_LLU_A_kallisto**	0.69	0.54	0.49	0.68	0.70	0.69
**C1_LLU_A_rsem**	0.80	0.62	0.57	0.79	0.80	0.80
**ICELL8_PE_A_featureCounts**	0.68	0.41	0.41	0.39	0.45	0.36
**ICELL8_PE_A_kallisto**	0.83	0.58	0.41	0.41	0.44	0.40
**ICELL8_PE_A_rsem**	0.77	0.42	0.42	0.40	0.44	0.40
**ICELL8_SE_A_featureCounts**	0.81	0.57	0.41	0.41	0.43	0.39
**ICELL8_SE_A_rsem**	0.55	0.43	0.44	0.37	0.42	0.37
**ICELL8_SE_A_kallisto**	0.71	0.53	0.42	0.41	0.44	0.43

## Data Availability

Reference datasets are available from scrnabench package: https://github.com/NWhitener/scrnabench, accessed on 1 December 2022. Synthetic datasets and code are available at https://github.com/SheltonZhaoK, accessed on 1 December 2022.

## Abbreviations

The following abbreviations are used in this manuscript:

ARI	Adjusted Rand Index
DBI	Davies Bouldin Index
GA	Genetic Algorithm
ML	Machine Learning
MOGA	Multi-Objective Genetic Algorithm
MOO	Multi-Objective
MSE	Mean Square Error
NDSS	Non-dominance Solution Sorting
NMI	Normalized Mutual Information
Sil	Silhouette Coefficient
SOGA	Single Objective Genetic Algorithm
